# Emerging Coxsackievirus A6 Causing Hand, Foot and Mouth Disease, Vietnam

**DOI:** 10.3201/eid2404.171298

**Published:** 2018-04

**Authors:** Nguyen To Anh, Le Nguyen Truc Nhu, Hoang Minh Tu Van, Nguyen Thi Thu Hong, Tran Tan Thanh, Vu Thi Ty Hang, Nguyen Thi Han Ny, Lam Anh Nguyet, Tran Thi Lan Phuong, Le Nguyen Thanh Nhan, Nguyen Thanh Hung, Truong Huu Khanh, Ha Manh Tuan, Ho Lu Viet, Nguyen Tran Nam, Do Chau Viet, Phan Tu Qui, Bridget Wills, Sarawathy Sabanathan, Nguyen Van Vinh Chau, Louise Thwaites, H. Rogier van Doorn, Guy Thwaites, Maia A. Rabaa, Le Van Tan

**Affiliations:** Oxford University Clinical Research Unit, Ho Chi Minh City, Vietnam (N.T. Anh, L.N.T. Nhu, H.M.T. Van, N.T.T. Hong, T.T. Thanh, V.T.T. Hang, N.T.H. Ny, L.A. Nguyet, B. Wills, S. Sabanathan, L. Thwaites, H.R. van Doorn, G. Thwaites, M.A. Rabaa, L.V. Tan);; Hospital for Tropical Diseases, Ho Chi Minh City (T.T.L. Phuong, P.T. Qui, N.V.V. Chau);; Children’s Hospital 1, Ho Chi Minh City (L.N.T. Nhan, N.T. Hung, T.H. Khanh);; Children’s Hospital 2, Ho Chi Minh City (H.M. Tuan, H.L. Viet, N.T. Nam, D.C. Viet);; University of Oxford, Oxford, United Kingdom (L. Thwaites, H.R. van Doorn, G. Thwaites, M.A. Rabaa)

**Keywords:** Hand foot and mouth disease, Vietnam, deep sequencing, phylogeny, coxsackievirus A6, Asia, enteroviruses, viruses

## Abstract

Hand, foot and mouth disease (HFMD) is a major public health issue in Asia and has global pandemic potential. Coxsackievirus A6 (CV-A6) was detected in 514/2,230 (23%) of HFMD patients admitted to 3 major hospitals in southern Vietnam during 2011–2015. Of these patients, 93 (18%) had severe HFMD. Phylogenetic analysis of 98 genome sequences revealed they belonged to cluster A and had been circulating in Vietnam for 2 years before emergence. CV-A6 movement among localities within Vietnam occurred frequently, whereas viral movement across international borders appeared rare. Skyline plots identified fluctuations in the relative genetic diversity of CV-A6 corresponding to large CV-A6–associated HFMD outbreaks worldwide. These data show that CV-A6 is an emerging pathogen and emphasize the necessity of active surveillance and understanding the mechanisms that shape the pathogen evolution and emergence, which is essential for development and implementation of intervention strategies.

Hand, foot and mouth disease (HFMD) is an emerging infection that has overwhelmed countries in the Asia–Pacific region over the past 2 decades. The outbreak in Sarawak, Malaysia, in 1997 caused 2,628 reported cases and 29 deaths and marked the start of explosive regional HFMD outbreaks in subsequent years. On average, >1 million cases have been recorded in China annually since 2008 (*1*). In Vietnam, the average annual incidence is ≈80,000 cases; an epidemic peak occurred during 2011–2012, resulting in >200,000 cases and >200 deaths (*2*).

HFMD is caused by enterovirus A (genus *Enterovirus*, family *Picornaviridae*), but the epidemic patterns until now have been punctuated by the frequent replacement of dominant pathogens between enterovirus serotypes over time. Enterovirus A71 (EV-A71) and coxsackievirus A16 (CV-A16) have been regarded as the major causes of HFMD (*3*). CV-A6 was isolated in the United States in 1949 (*4*) and has steadily become one of the main viruses causing HFMD outbreaks in Europe, America, and Asia, including China, Japan, Taiwan, and Thailand (*3*,*5*–*10*). Unlike EV-A71, for which the (sub)genogroup designation has been well established (*11*), but similar to other coxsackieviruses (CV-A16 and CV-A10), CV-A6 is arbitrarily divided into several phylogenetic clusters or lineages, from cluster A to F (*12*) or lineage A to E (E1 and E2) (*3*). Cluster A/lineage E2 is distributed worldwide and has frequently been detected in recent outbreaks. We use the term cluster in this article.

Despite the public health burden of HFMD, no antiviral drug has been clinically proven effective. A vaccine for EV-A71 has recently been licensed in China only (*13*), and CV-A16 vaccines (either monovalent or EV-A71/CV-A16 bivalent forms) are under development (*14*,*15*).

The emergence of CV-A6 has further challenged the development of intervention strategies, including vaccines, to reduce the burden of HFMD (*13*). It also emphasizes the need to better understand the molecular evolution of this emerging pathogen, which is essential for development of an effective CV-A6 vaccine in the future (*16*). However, few studies from endemic countries have documented the longitudinal evolution of CV-A6 (*5*,*17*–*19*). In this study, we applied next-generation sequencing to obtain whole-genome sequences for CV-A6 strains sampled from primary, secondary, and tertiary referral hospitals in Ho Chi Minh City, Vietnam, during 2011–2015. To investigate the molecular evolution and recent spread of CV-A6, we performed phylogenetic and phylogeographic analysis on both a global scale and within the southern provinces of Vietnam.

## Materials and Methods

### Patients and Clinical Samples

We obtained the clinical samples used in this study from patients enrolled in an HFMD research program in which outpatients and inpatients with all severities of disease were recruited (*20*). During August 2011–June 2013, we carried out the research program at the pediatric intensive care unit (PICU) of the Hospital for Tropical Diseases in Ho Chi Minh City, Vietnam. This PICU admitted only patients with severe HFMD (the clinical grading system is described in the Technical Appendix). In the subsequent phase (July 2013–December 2015), we expanded patient enrollment to outpatient clinics, infectious disease wards, and PICUs in 3 major referral hospitals in Ho Chi Minh City (Children’s Hospital 1, Children’s Hospital 2, and Hospital for Tropical Diseases). We selected for analysis CV-A6–positive throat and rectal swab specimens with sufficient viral load (samples with real-time PCR crossing point values of <30 [*21*]) collected in viral transport medium from study participants. The real-time reverse transcription PCR (RT-PCR) methods used are described in the Technical Appendix.

### CV-A6 Whole-Genome Sequencing and Sequence Assembly

We performed whole-genome sequencing of CV-A6 on the selected swabs with sufficient viral load using a previously described MiSeq-based approach (*21*). In brief, we pretreated 110 μL of selected swab specimens in viral transport medium by a centrifugation step at 13,000 rpm for 10 min to remove host cells or large cellular components and followed this step with DNase treatment of the obtained supernatants. We then isolated viral nucleic acid (NA) using a QIAamp viral RNA kit (QIAGEN, Hilden, Germany) and recovered it in 50 μL of the elution buffer (provided with the kit). We subjected 10 μL of the isolated NA to cDNA synthesis using a Super Script III kit (Invitrogen, Carlsbad, CA, USA) and FR26RV-Endoh primer (*21*). We then converted the cDNA to double-stranded DNA using exo-Klenow (Invitrogen) and preamplified the cDNA using Platinum PCR supermix (Invitrogen) and FR20RV primer (*21*). We then purified the PCR product and subjected it to library preparation using a Nextera XT DNA sample preparation kit (Illumina, San Diego, CA, USA). Finally, we sequenced the product using MiSeq reagent kits (Illumina) in a MiSeq platform (Illumina) (*21*).

We performed whole-genome sequence assembly using the Geneious 8.1.5 software package (Biomatters, Auckland, New Zealand) with a reference-based mapping approach. This method involves the mapping of individual reads of each sample to a reference sequence and manual editing of the consensus.

### Multiple Sequence Alignment, Recombination Detection, and Phylogenetic Analysis

We performed multiple sequence alignment using MUSCLE (multiple sequence comparison by log-expectation) (*22*), available in Geneious. For Vietnam sequences, we then calculated the percentages of sequence identities among them from the resulting multiple sequence alignment files using Geneious.

We inferred recombination using a combination of methods (Chimera, GENECONV, Maxchi, Bootscan, and Siscan) within RDP4 (Recombination Detection Program version 4) (*22*) using the default settings with recombination supported if >3 methods showed significant values (p<0.05) and reconfirmed findings by phylogenetic analysis. We then removed identified recombinants from further phylogenetic analysis.

To investigate the relationship between Vietnam CV-A6 strains and global strains downloaded from GenBank, we constructed maximum-likelihood trees for viral capsid protein 1 (VP1) and complete coding sequences (CDS) using IQ-TREE version 1.4.3 (*23*). The maximum likelihood phylogenetic analysis used the general time reversible (for CDS dataset) and Tamura-Nei 93 (for VP1 dataset) nucleotide substitution models with a gamma distributed among site rate variation (4 rate categories). We assessed support for individual nodes using a bootstrap procedure (10,000 replicates).

We analyzed the phylogeographic history of CV-A6 in Vietnam and worldwide using BEAST version 1.8.3 (https://github.com/beast-dev/beast-mcmc/releases/tag/v1.8.3). We performed this analysis for both complete CDS and VP1 sequences downloaded from GenBank (October 2016) and the sequences obtained from the present study. For GenBank sequences, we excluded all the partial sequences, identical sequences, sequences with internal gaps, recombinant sequences, and sequences without sampling dates or locations. We then performed regression analysis implemented in TempEst (https://academic.oup.com/ve/article/2/1/vew007/1753488) to further exclude sequences with insufficient temporal signals. For global strains, 170 VP1 and 52 complete CDS sequences from China, Finland, France, India, Japan, Spain, Taiwan, and the United Kingdom were included for analysis. For Vietnam sequences, we used 98 sequences. Southern provinces in Vietnam from where the viruses were sampled were grouped into 3 discrete locations: Ho Chi Minh City (from where about half of the HFMD cases from Vietnam have been reported), southeast provinces (Long An, Can Tho, Tien Giang, Kien Giang, Dong Thap, and Hau Giang provinces), and Mekong Delta provinces (Tay Ninh, Dong Nai, Binh Duong, Binh Phuoc, Ba Ria, and Vung Tau provinces). Small sample sizes from individual provinces precluded phylogeographic analyses at a finer spatial scale.

For all analyses, we used the general time reversible (*24*) (for the CDS dataset) and Tamura-Nei 93 (*25*) (for the VP1 dataset) nucleotide substitution models with a gamma distributed among site rate variation (4 rate categories) (as indicated by IQ-TREE), the strict molecular clock model, and a Bayesian skyline plot (10 groups). We employed a Bayesian Markov chain Monte Carlo framework (available in BEAST) with 800 million steps and sampling every 80,000 steps. We assessed convergence using Tracer version 1.5 (http://tree.bio.ed.ac.uk/software/tracer/). We selected a burn-in threshold of 10% and accepted effective sample size values above 200. Maximum-clade credibility (MCC) trees were then summarized with TreeAnnotator (available in the BEAST package) and visualized in Figtree version 1.4.2 (http://tree.bio.ed.ac.uk/software/figtree**)**.

To estimate the relative genetic diversity of CV-A6 over time, we analyzed CDS and VP1 sequences separately using the same Bayesian skyline method. We submitted the sequences of CV-A6 obtained in this study to the National Center for Biotechnology Information (GenBank accession nos. MF578282–MF578381).

### Ethics Considerations

The study was approved by the corresponding institutional review board of the local hospitals in Vietnam where patients were enrolled: Children’s Hospital 1, Ho Chi Minh City; Children’s Hospital 2, Ho Chi Minh City; and Hospital for Tropical Diseases, Ho Chi Minh City. The study was also approved by the Oxford Tropical Research Ethics Committee and was performed in accordance with the ethics standards noted in the 1964 Declaration of Helsinki and its later amendments, or comparable ethics standards.

## Results

### Baseline Characteristics of Patients with CV-A6 Infections

**Figure 1:**
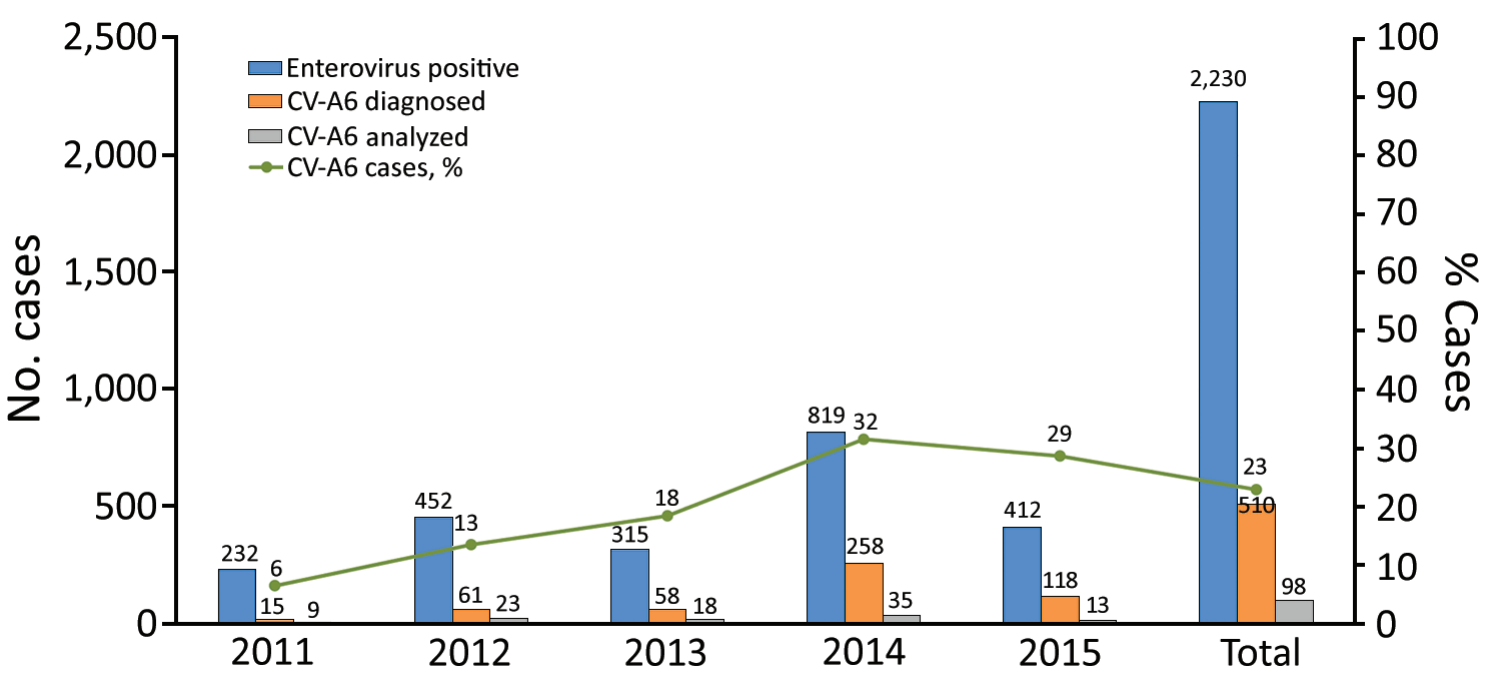
Temporal distribution of PCR-positive hand, foot and mouth disease cases and detection rates of CV-A6 during 2011–2015, Vietnam. CV, coxsackievirus.

During August 2011–December 2015, a total of 514 patients with HFMD had specimens that tested positive for CV-A6, accounting for 23% of the HFMD study participants who were enterovirus PCR positive (n = 2,230). We detected EV-A71 in 36% (812) of the patients, CV-A16 in 10% (240), and CV-A10 in 7% (164). Temporally, the detection rate of CV-A6 in HFMD patients increased from 6% in 2011 to 13% in 2012, 18% in 2013, 32% in 2014, and 29% in 2015 (Figure 1). Complete data on demographics and clinical grades were available from 510/514 CV-A6 infected patients (Table). Although CV-A6–associated HFMD was mostly mild, 93 (18%) patients had grade 2b1, 2b2, or 3 HFMD (i.e., severe HFMD), accounting for 76/76 (100%) of patients with CV-A6 who were enrolled in the first phase of the study and 17/434 (4%) of patients with CV-A6 who were enrolled in the second phase of the study.

**Table:** Demographics and clinical severity grades for patients with CV-A6–associated HFMD, Vietnam, 2011–2015*

Demographics	Total, n = 510	2011–2012, n = 76	2013–2015, n = 434	Group 1, patients with CDS included in analyses, n = 98	Group 2, patients excluded from phylogenetic analyses, n = 412
Sex					
M	330 (64.7)	50 (65.8)	280 (64.5)	68 (69.4)	262 (63.6)
F	180 (35.3)	26 (34.2)	154 (35.5)	30 (30.6)	150 (36.4)
Age, mo					
Median	16.07	15.23	16.18	15.52	16.17
IQR	11.57–22.46	9.83–24.74	11.85–22.43	10.68–24.48	11.71–22.41
Highest grade					
1	188 (36.8)	0	188 (43.3)	39 (39.8)	149 (36.1)
2a	229 (44.9)	0	229 (52.8)	26 (26.5)	203 (49.3)
2b1	81 (15.9)	68 (89.5)	13 (3.0)	30 (30.6)	51 (12.4)
2b2	5 (1.0)	2 (2.6)	3 (0.7)	1 (1.0)	4 (1.0)
3	7 (1.4)	6 (7.9)	1 (0.2)	2 (2.0)	5 (1.2)
Location					
HCMC	327 (64.1)	54 (71.1)	273 (62.9)	57 (58.2)	270 (65.5)
Mekong Delta†	89 (17.5)	17 (22.4)	72 (16.6)	21 (21.4)	68 (16.5)
Southeast‡	88 (17.3)	5 (6.6)	83 (19.1)	20 (20.4)	68 (16.5)
Others§	6 (1.1)	0	6 (1.4)	0	6 (1.5)

*Values are no. (%) patients except as indicated. CV, coxsackievirus; HCMC, Ho Chi Minh City; HFMD, hand, foot and mouth disease; IQR, interquartile range. †Long An, Can Tho, Tien Giang, Kien Giang, Dong Thap, and Hau Giang provinces. ‡Tay Ninh, Dong Nai, Binh Duong, Binh Phuoc, Ba Ria, and Vung Tau provinces. §Binh Thuan (n = 2), Hai Duong (n = 1), Quang Ngai (n = 1), Khanh Hoa (n = 1), and Thanh Hoa (n = 1).

### CV-A6 Whole-Genome Sequences

From the 514 patients who had CV-A6, we subjected 131 swabs (97 throat swabs and 34 rectal swabs) with sufficient viral load to whole-genome sequencing. Of these, we successfully recovered 100 nearly complete or complete coding sequences (80%). We identified evidence of recombination in 2 CV-A6 sequences (data not shown) and removed these 2 sequences from subsequent phylogenetic analyses.

### Phylogeny and Phylogeography

**Figure 2:**
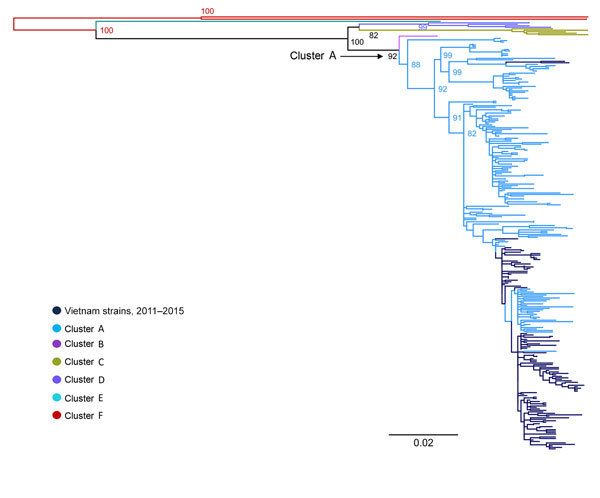
Maximum-likelihood tree of viral capsid protein 1 sequences of coxsackievirus A6 strains from Vietnam and worldwide. Branches are colored by cluster; cluster A, which includes the Vietnam strains, is indicated. Scale bar indicates nucleotide substitutions per site.

Phylogenetic analyses of 282 VP1 sequences of global strains, including 98 from Vietnam, showed that CV-A6 was grouped into 6 genetic clusters, in agreement with a previous report (*12*). All of the Vietnam CV-A6 isolates belonged to cluster A (Figure 2), showing nucleotide identity of 91.8%–100% and amino acid identity of 98.6%–100%. This genogroup consists of viruses sampled from various geographic locations worldwide, whereas the CV-A6 strains from Vietnam fell within a viral lineage consisting of CV-A6 strains from China, India, Japan, Taiwan, and the United Kingdom.

**Figure 3:**
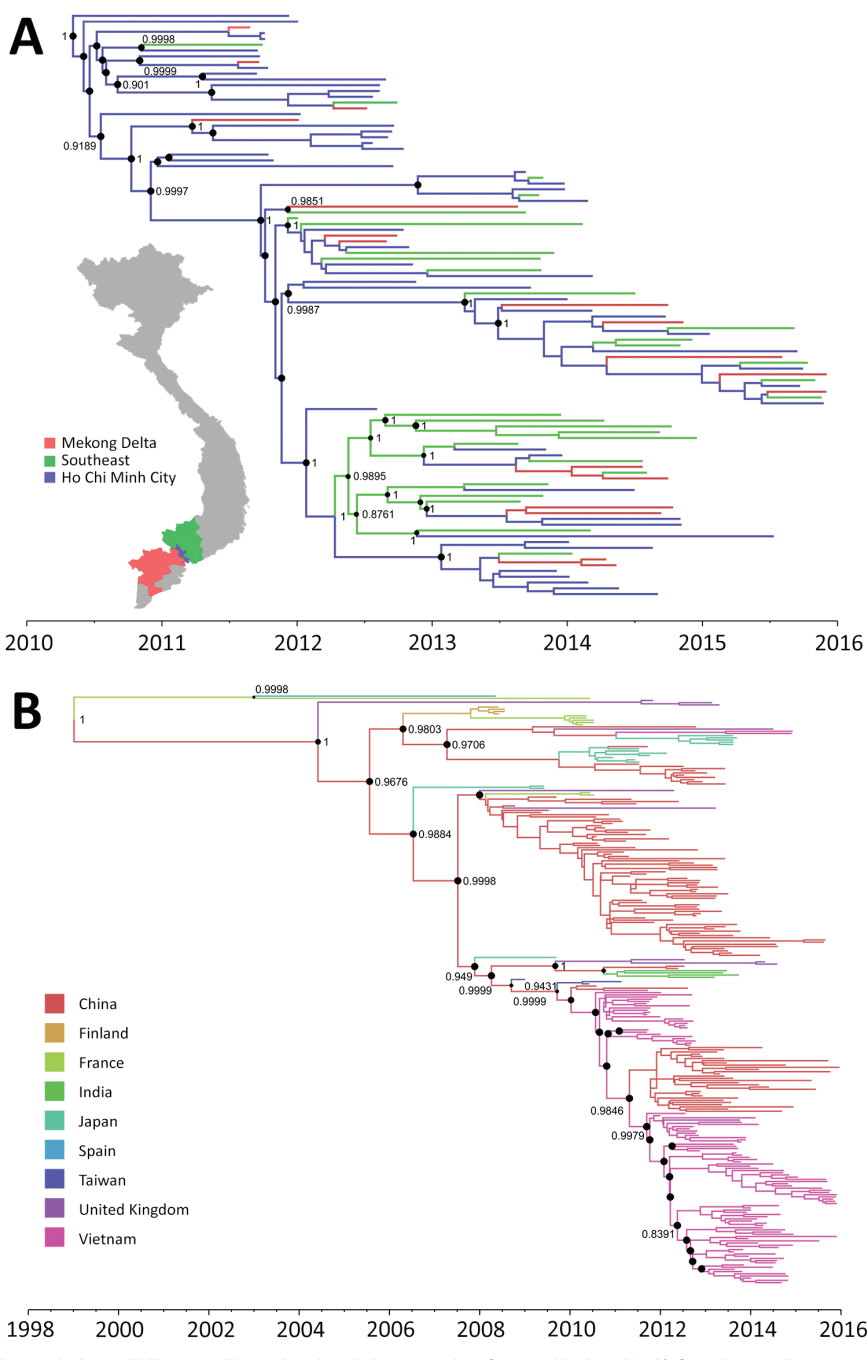
Maximum clade credibility trees illustrating the phylogeography of coxsackievirus A6. A) Complete coding sequence–based tree of Vietnam strains; B) viral capsid protein 1–based tree of global strains. Branches are color-coded according to location of sampling. Posterior probabilities >85% and state probabilities >75% (black circles) are indicated at all nodes. Map in panel A obtained from https://mapchart.net.

Delineating the dispersal of an emerging pathogen between geographic locations and within endemic countries is critical for outbreak control. In-depth phylogeographic analysis for all 13 discrete provinces from where the patients came was uninformative because of the small sample sizes from some provinces. When we conducted the analysis for 3 main discrete geographic locations, the results revealed that CV-A6 spread widely within southern Vietnam during the sampling period (Figure 3, panel A; Technical Appendix Figure 1, panel A). This finding is in contrast with what has been observed at the international level, at which movement of CV-A6 between endemic countries appears rare (Figure 3, panel B; Technical Appendix Figure 1, panel B).

Estimation of the time to the most recent common ancestor in the phylogeny including only strains from Vietnam suggested that the CV-A6 lineage that circulated in Vietnam during this time period began circulating within the country by 2010. This estimation is consistent between VP1-based and complete CDS-based analyses, which show time to most recent common ancestor estimates of November 2010 (95% CI May 2010–March 2011) and May 2010 (95% CI February 2010–August 2010), respectively. These estimates suggest that CV-A6 was being transmitted in Vietnam for at least 2 years before becoming the dominant cause of HFMD in 2012. The nucleotide substitution rate of VP1 sequences was estimated to be 7.42 × 10^–3^ (95% CI 6.1126 × 10^–3^ to 8.722 × 10^–3^) and the nucleotide substitution rate of complete CDS was estimated to be 4.556 × 10^–3^ (95% CI 4.209 × 10^–3^ to 4.913 × 10^–3^) substitutions per site per year. 

### CV-A6 Demographics

**Figure 4:**
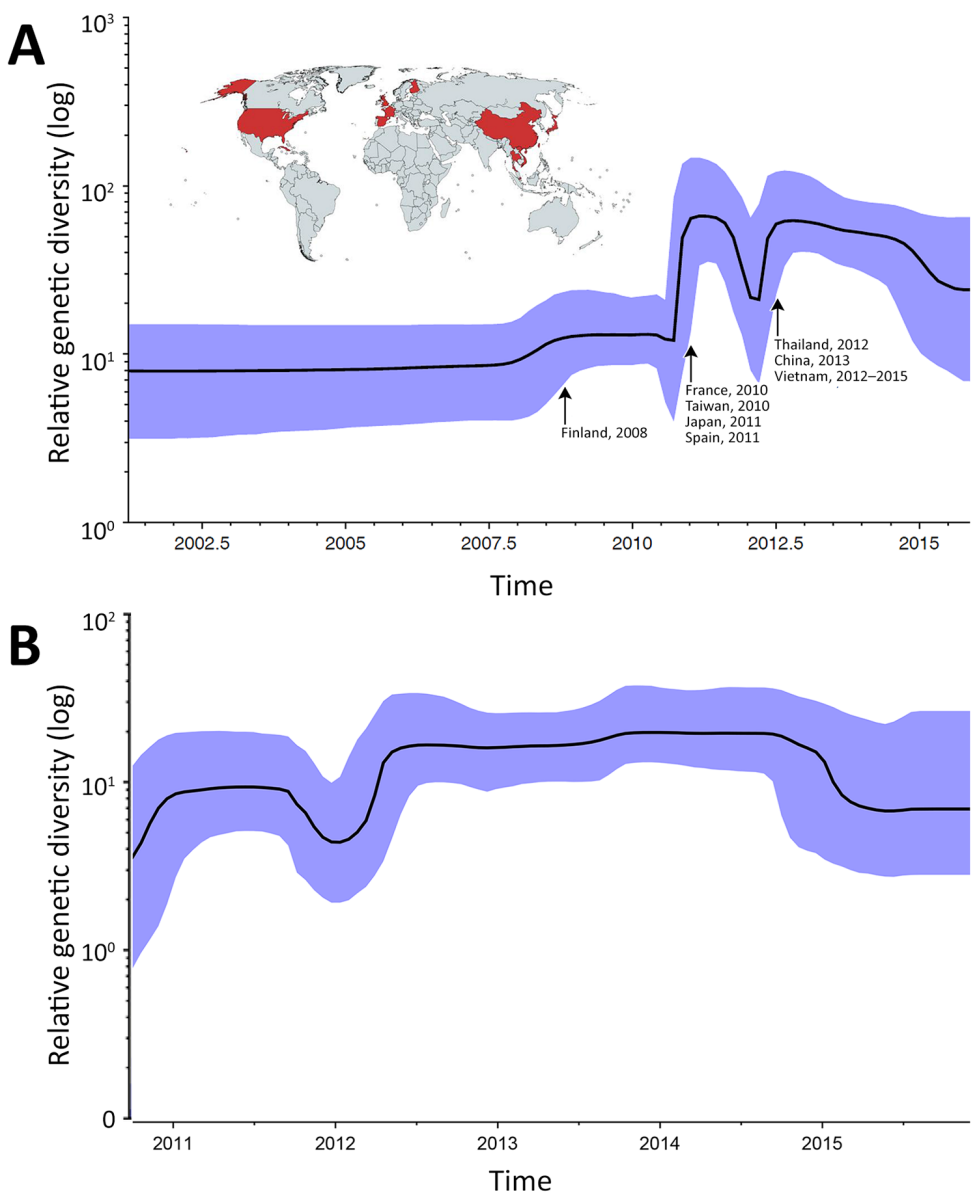
Skyline plots depicting the relative genetic diversity of CV-A6 over time. A) Result obtained from the analysis of viral capsid protein 1 sequences of global strains; B) result obtained from the analysis of complete coding sequences of Vietnam strains. Blue shading indicates 95% highest posterior density interval. Arrows in panel A indicate worldwide CV-A6 outbreaks and associated fluctuations in relative genetic diversity ; map (obtained from https://mapchart.net) illustrates the countries in which CV-A6–associated HFMD outbreaks have been recorded to date (*3*). No sequences from Cuba, Singapore, or the United States fulfilled the selection criteria for the skyline plot and phylogenetic analyses (see Methods section). CV, coxsackievirus.

Because of the relatively small number of CV-A6 whole genome sequences available in GenBank, we first assessed the global demographic history of the lineage using skyline plot analysis on VP1 sequences. The Bayesian VP1-based skyline plot of genogroup A viruses sampled across the world revealed fluctuations in the relative genetic diversity of CV-A6 from 2008 onward, especially during 2010–2012 (Figure 4, panel A), highlighting notable changes in viral diversity. This phenomenon coincided with CV-A6 outbreaks reported worldwide, including the 2008 Finland outbreak and major outbreaks affecting Asia, Europe, and the United States in subsequent years.

Bayesian skyline plot analysis for the CDS of the Vietnamese viruses alone did not suggest any major changes in relative genetic diversity during 2011–2015 compared with a global scale (Figure 4, panel B). Similar results were obtained when the analyses were done for VP1 sequences of the Vietnam strains and CDS of global strains (Technical Appendix Figure 2).

## Discussion

We report the evolutionary process of CV-A6 in Vietnam during 2011–2015. In addition, we summarize its detection rate in HFMD patients and associated demographics and clinical outcomes. Of 510 patients with CV-A6 infection, 93 (18%) had severe HFMD (grade 2b1 or above). However, 18% should not be interpreted as the proportion of CV-A6 infections associated with severe disease because most severe cases (76/93 [82%]; Table) came from the first phase of the study, during which only patients with severe HFMD were enrolled. However, when patients with all severities of HFMD were enrolled in the second phase of the study, CV-A6–associated severe HFMD accounted for 4% (17/434; Table) of the total number of CV-A6 patients. This result is in accordance with previous reports showing that HFMD is a mild disease with sporadic severe cases and demonstrates the potential association of CV-A6 with severe HFMD (*26*).

Our analysis placed the Vietnam CV-A6 strains within cluster A of CV-A6. This cluster A includes viruses sampled from various countries worldwide, and has emerged only recently: in Finland in 2008, followed by France, the United Kingdom, and subsequently the United States and countries in Asia in subsequent years (*3*,*5*,*6*,*8*–*10*,*18*). Although this migration highlights the global dispersal of cluster A viruses, and their potential to cause outbreaks worldwide, phylogeographic analysis of global strains did not reveal a high frequency of CV-A6 movement between endemic countries. In contrast, viral transmission between geographic locations within southern Vietnam appeared frequently; Ho Chi Minh City is a likely source of viral circulation given the observed phylogeographic patterns, and transmission is highly connected with other endemic countries in the region and southern provinces in Vietnam through international and domestic transport. This observation is in agreement with previously observed patterns of EV-A71 transmission within and between endemic countries (*20*,*27*). 

HFMD affects mostly young children, especially those <5 years of age, and humans are the only known natural host of HFMD-causing enteroviruses. It is therefore likely that human movement is critical to the transmission and spread of HFMD at both global and local scales. Although young children likely play a major role in local transmission of these pathogens, asymptomatic adults carrying HFMD-causing viruses have previously been reported (*28*); because adults are more likely to travel longer distances, they may play an integral role in the movement of these viruses across long distances.

Dating analysis based on VP1 and CDS consistently showed that CV-A6 had been circulating in Vietnam for 2 years before it emerged as a cause of illness in 2012 and subsequently become a dominant pathogen of HFMD. This finding parallels previous studies showing that emerging EV-A71 subgenogroups circulated cryptically for 2–3 years before emergence (*20*,*27*). Likewise, although our estimated evolutionary rates of CV-A6 were slightly different between VP1 and complete CDS, this finding is likely because CV-A6 VP1 is the main target of neutralizing antibody (*29*) and is therefore subjected to a higher selection pressure than other viral proteins. Still, our findings were within the ranges of previous estimations for emerging enteroviruses such as EV-A71 (*27*).

Skyline plots generated using global strains illustrate that CV-A6 cluster A maintained a relatively constant population size until 2010–2011, when a sharp increase in relative genetic diversity was detected along with outbreaks in several countries. Notably, although the most recent common ancestor for the Vietnam CV-A6 lineage is inferred to have existed during the first half of 2010, the first CV-A6 infections found in this study were in patients enrolled in September 2011. The skyline plot did not reveal major changes in terms of genetic diversity of the Vietnam CV-A6 strains during the study period, which may suggest that large-scale transmission occurred in the community for some months before its detection in hospitals.

The observed demographic features in this study should be interpreted with caution. For the Vietnam CV-A6 strains, the discordance between the fluctuating numbers of CV-A6 infections detected per year and the relatively constant demographic picture illustrated by the skyline plot may be the result of sampling bias inherent to the study, the initial focus of which was enrollment of patients with severe enough illness to go to the pediatric ICU; only later expansion included patients at outpatient facilities. Viruses of the family *Picornaviridae* (including CV-A6) are ubiquitous, and most infections are asymptomatic. It is therefore possible that the proportion of CV-A6 viruses detected during the first half of this study may have underestimated the overall epidemiologic burden of CV-A6 relative to other HFMD-causing enteroviruses during this time period because of the focus on severe cases, given that CV-A6 is proportionally less likely to cause severe disease than EV-A71. Likewise, for global strains, the skyline plot analysis in this study was in part based on publicly available CV-A6 sequences derived from studies across the world, of which most were from recent epidemic years (Technical Appendix Figure 3); as such, the dramatic fluctuations in relative genetic diversity shown on the skyline plot in highly sampled epidemic years may be partly attributed to this sampling bias. However, the general trend toward increasing genetic diversity shown in the skyline plot during 2002–2015 would not be strongly affected by this bias and is likely to reflect a true increase in CV-A6 infections and genetic diversity during this period.

Together, these data emphasize the importance of active surveillance for molecular epidemiology of HFMD in disease-endemic countries. It is also critical to identify the underlying mechanisms that shape the evolutionary process and the emergence of new HFMD-causing enterovirus lineages in countries with high HFMD endemicity. Further research in these key areas would have profound implications for the development and implementation of HFMD vaccines. We hypothesize that population immunity and antigenic differences between circulating strains and emergent lineages are key drivers of the transmission dynamics and epidemiology of HFMD; therefore, studies to characterize cross-neutralization titers in serum samples of patients infected with common serotypes, including EV-A71, CV-A6, CV-A10, and CV-A16, to inform vaccine development are needed and ongoing.

Technical Appendix Additional information on the clinical grading system for hand, foot and mouth disease and the procedure for detection of enteroviruses in clinical samples, Vietnam.
