# Supplementary material for: Emerging Coxsackievirus A6 Causing Hand, Foot and Mouth Disease, Vietnam
Source: Emerg Infect Dis. 2018 Apr;24(4):654–62. doi: 10.3201/eid2404.171298 (PMC5875260; doi:10.3201/eid2404.171298)
Supplement: Technical Appendix — Additional information on the clinical grading system for hand, foot and mouth disease and the procedure for detection of enteroviruses in clinical samples, Vietnam. [file 17-1298-Techapp-s1.pdf]

Article DOI: https://doi.org/10.3201/eid2404.171298

Emerging Coxsackievirus A6 Causing Hand, Foot and Mouth Disease, Vietnam

Technical Appendix

Clinical Grading System for HFMD (*1*)

• Grade 1: mouth ulcers or vesicles/papules on hands, feet, or buttocks, with or without mild fever (<39°C)

• Grade 2a: central nervous system (CNS) involvement (myoclonus reported by parents or caregivers only, fever >39°C or ataxia)

• Grade 2b1: myoclonus observed by medical staff or history of myoclonus and lethargy or pulse higher than 130 bpm

• Grade 2b2: ataxia, nystagmus, limb weakness, cranial nerve palsies, persistent high fever, or pulse higher than 150 bpm

• Grade 3: autonomic dysfunction with sweating, hypertension, tachycardia, and tachypnea

• Grade 4: additional cardiopulmonary compromise with pulmonary edema or shock syndrome

Enterovirus Real-Time RT-PCR

The procedure for detection of enteroviruses in clinical samples was carried out as previously described (*2*), and is detailed as follows.

We extracted viral RNA from throat and rectal swabs using the QIAamp Viral RNA Mini kit (QIAGEN, Hilden, Germany). In brief, we first mixed 140 µL of throat/rectal swabs in viral transport medium with 20 µL of equine arteritis virus and then extracted total nucleic acid

according to the manufacturer’s instructions, eluted it in 100 μL elution buffer and stored it at –80°C until used.

We performed real-time RT-PCR using the SuperScript III One-Step qRT-PCR System with Platinum Taq DNA Polymerase (Invitrogen, Carlsbad, CA, USA) in a LightCycler 480 II machine (Roche Diagnostics, Mannheim, Germany). The reaction was performed in a final volume of 25 μL containing 12.5 μL 2X RT-PCR reaction Mix (Invitrogen), primers and probes at appropriate concentrations (Technical Appendix Table), 0.5 μL enzyme mix, and 2 μL viral RNA. The cycling conditions included 1 cycle of 60°C for 3 minutes, followed by 15 minutes at 53°C and 2 minutes at 95°C, and 45 cycles of 15 seconds at 95°C, 1 min at 53°C (including fluorescence acquisition), and 15 seconds at 72°C.

References

1. Khanh TH, Sabanathan S, Thanh TT, Thoa PK, Thuong TC, Hang V, et al. Enterovirus 71-associated hand, foot, and mouth disease, Southern Vietnam, 2011. Emerg Infect Dis. 2012;18:2002–5. http://dx.doi.org/10.3201/eid1812.120929

2. Thanh TT, Anh NT, Tham NT, Van HM, Sabanathan S, Qui PT, et al. Validation and utilization of an internally controlled multiplex real-time RT-PCR assay for simultaneous detection of enteroviruses and enterovirus A71 associated with hand foot and mouth disease. Virol J. 2015;12:85. http://dx.doi.org/10.1186/s12985-015-0316-2

Technical Appendix Table. Primer and probe sequences and final concentrations used in 1 reaction for detecting enterovirus in clinical samples*

Name	Sequence (5′→3′)	Final concentration	Notes
EAV-F primer	CATCTCTTGCTTTGCTCCTTA G	400 nM	Internal control
EAV-R primer	AGCCGCACCTTCACATTG	400 nM	
EAV-probe	FAM-CGCTGTCAGAACAACATTATTGCCCAC-BHQ1	100 nM	
ENT-F	CCCTGAATGCGGCTAAT	400 nM	Enterovirus specific primers and probe
ENT-R	ATTGTCACCATAAGCAGCC	400 nM	
ENTr-probe	Cy5-ACCCAAAGTAGTCGGTTCCG -BHQ3	200 nM	
EV-A71–634F	GGAGAACACAARCARGAGAAAGA	400 nM	Enterovirus 71 specific primers and probe
EV-A71–743R	ACYAAAGGGTACTTGGAYTTVGA	400 nM	
EV-A71-probe	Cyan500-TGATGGGCACDTTCTCRGTGCG-BHQ1	40 nM	

*BHQ = black hole quencher; Cy5 = cyanine 5; Cyan500 = cyan 500 NHS ester; FAM = Carboxyfluorescein; D = A, G, and T; R = A and G; V = A, C, and G; Y = T and C.

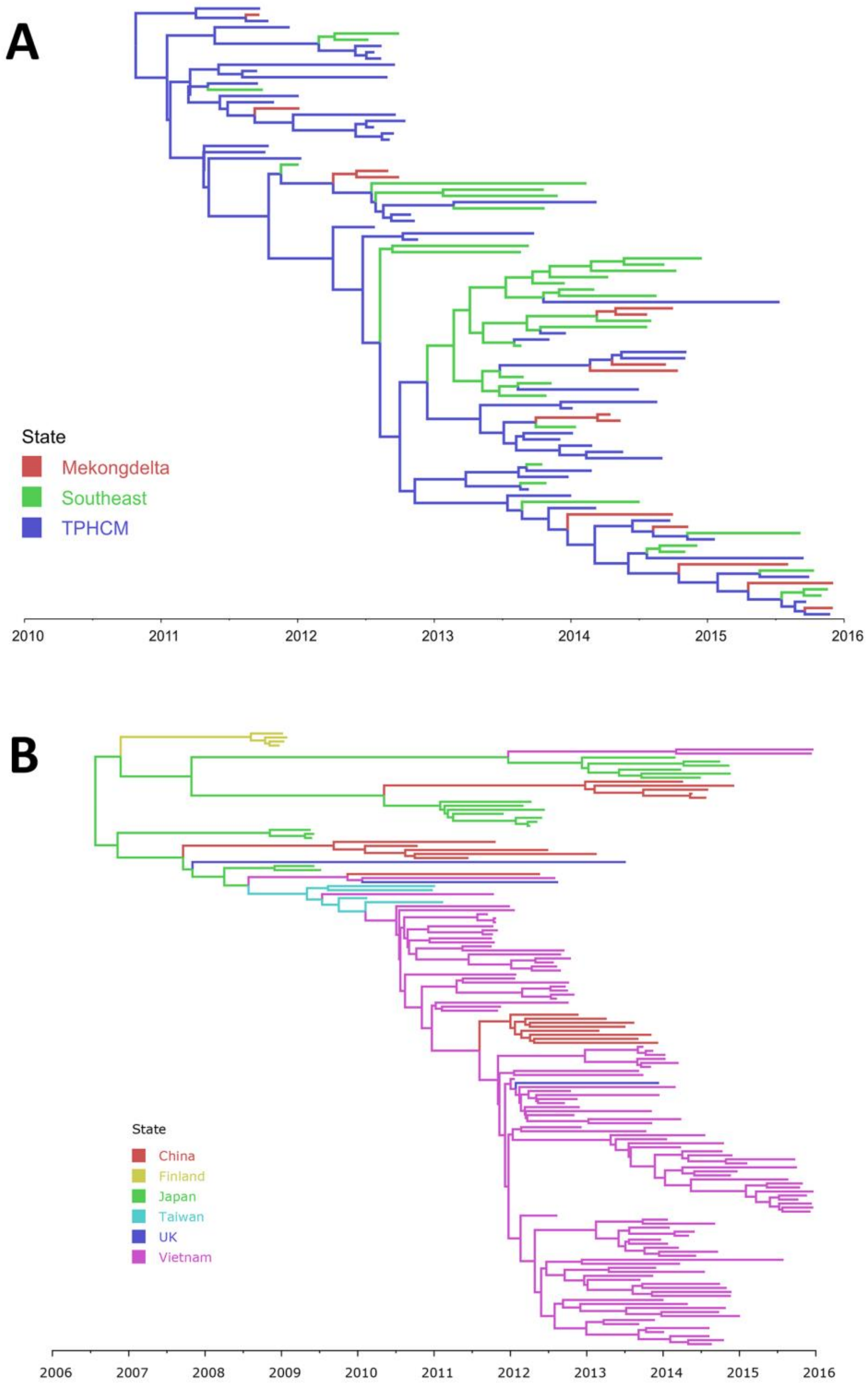

Technical Appendix Figure 1. Maximum clade credibility (MCC) trees illustrating the phylogeography of CV-A6: A) MCC tree of viral capsid protein (VP1) sequences of Vietnamese strains; B) MCC tree of complete coding sequences (CDS) of global strains. Branches are color-coded according to location of sampling.

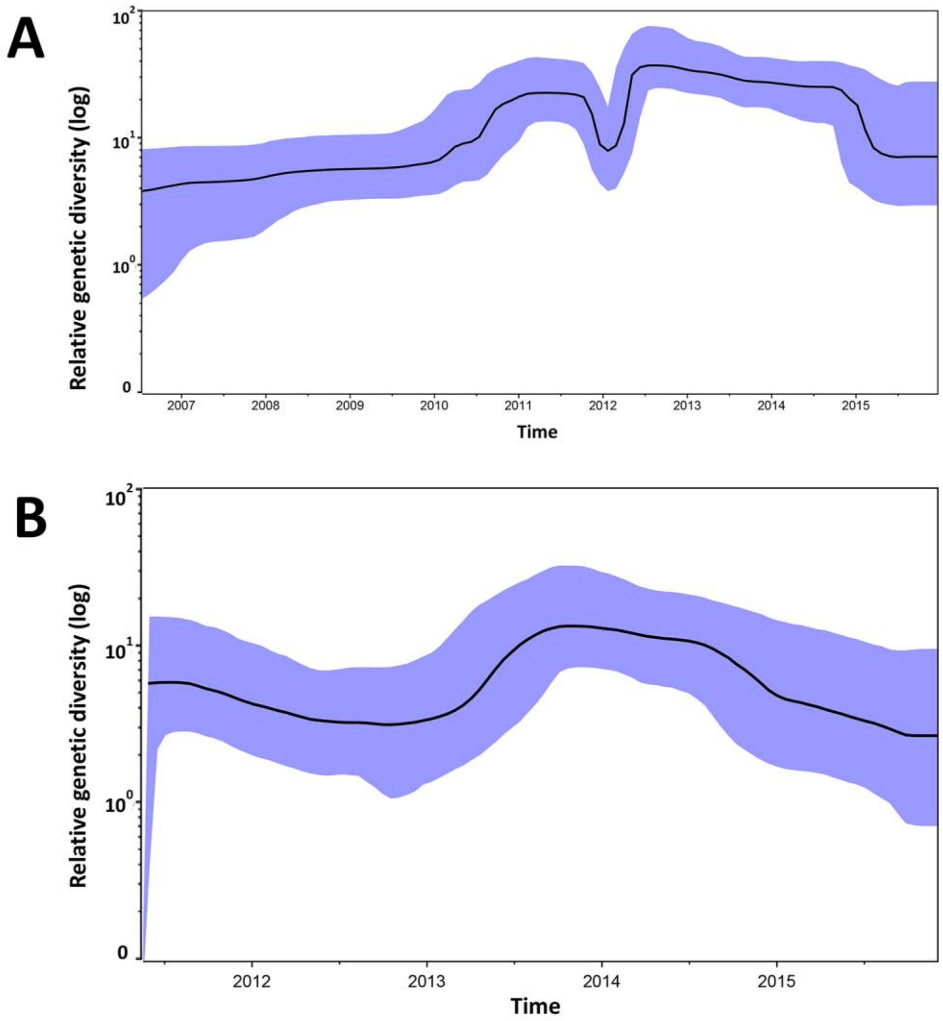

Technical Appendix Figure 2. Skyline plots depicting the relative genetic diversity of CV-A6 over time. A) Results obtained from the analysis of CDS of global strains; B) results obtained from the analysis of VP1 sequences of Vietnamese strains.

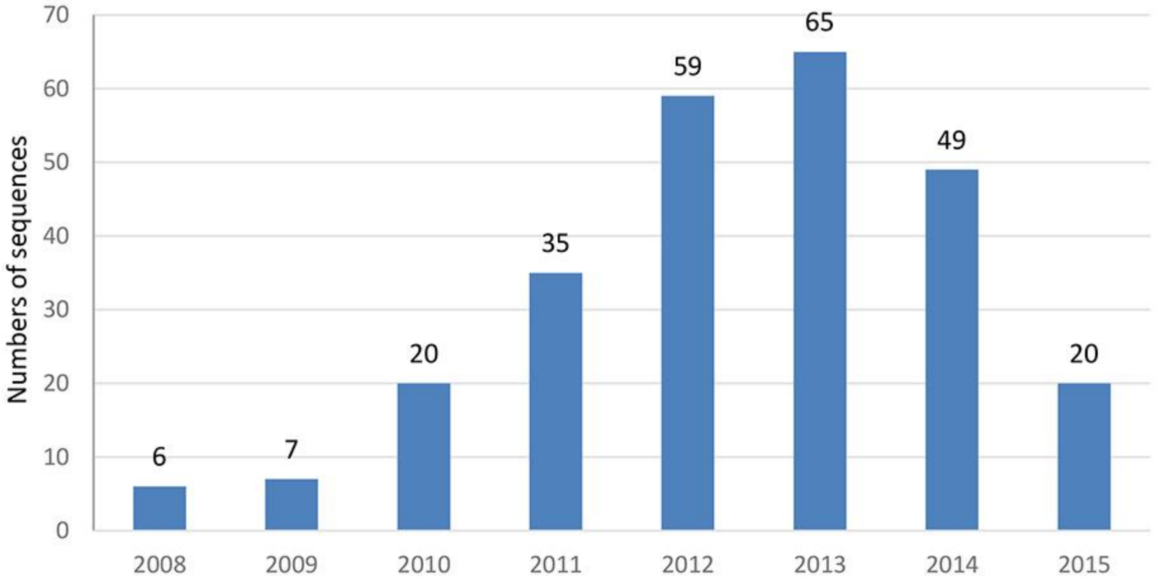

Technical Appendix Figure 3. Bar chart illustrating annual distribution of CV-A6 sequences used for phylogenetic analysis.
